# Prevalence of *bla*_*TEM-220*_ gene in Penicillinase-producing *Neisseria gonorrhoeae* strains carrying Toronto/Rio plasmid in Argentina, 2002 – 2011

**DOI:** 10.1186/s12879-015-1294-0

**Published:** 2015-12-16

**Authors:** Ricardo Gianecini, Claudia Oviedo, Cristina Guantay, Laura Piccoli, Graciela Stafforini, Patricia Galarza

**Affiliations:** Servicio de Enfermedades de Transmisión Sexual, Instituto Nacional de Enfermedades Infecciosas (INEI)-ANLIS “Dr. Carlos G. Malbrán”, Ciudad Autónoma de Buenos Aires, Argentina; Gonococcal Antimicrobial Susceptibility Surveillance Programme-Argentina (GASSP-AR), ᅟ, Argentina

**Keywords:** *Neisseria gonorrhoeae*, Beta-lactamase, TEM-220, Toronto/Rio plasmid, Argentina

## Abstract

**Background:**

Penicillinase**-**producing *Neisseria gonorroheae* (PPNG) was first isolated in 1976. PPNG strains carrying *bla*_TEM-1_ and *bla*_TEM-135_ gene have been described in different countries. Recently, a novel *bla*_TEM-220_ allele was detected in PPNG isolates carrying Toronto/Rio plasmid. The prevalence and characteristics of TEM-220 strains worldwide are unknown, and therefore, it needs to be studied. The purpose of this study was to detect *bla*_TEM-220_ gene in PPNG strains possessing Toronto/Rio plasmid over a period of ten years in Argentina, and to evaluate the proportion of isolates producing non-TEM-220 containing the T539C substitution in the *bla*_TEM_ allele.

**Methods:**

One hundred and fifty one PPNG isolates carrying Toronto/Rio plasmid were studied between 2002 and 2011. A mismatch amplification mutation assay (MAMA) PCR was used to identify the T539C substitution in the *bla*_TEM_ allele and a MAMA-PCR protocol was developed to detect the G547A substitution in the *bla*_TEM-220_. The reference agar dilution method of the Clinical and Laboratory Standard Institute (CLSI) was used for susceptibility testing to five β-lactams antibiotics, ciprofloxacin, tetracycline and azithromycin. In all TEM-220-producing isolates, the whole *bla*_TEM_ gene was sequenced and the isolates were typed using *N. gonorroheae* multiantigen sequence typing (NG-MAST).

**Results:**

MAMA PCR successfully identified the G547A substitution in the *bla*_TEM-220_ allele. The proportion of isolates that possessed the *bla*_TEM-220_ allele was 2.6 %, and 93.2 % MAMA TEM-220 PCR-negative isolates showed the T539C substitution in the *bla*_TEM_ gene. No differences in the susceptibility to five beta-lactam antibiotics tested were observed in PPNG isolates TEM-220-producing and PPNG isolates carrying the T539C substitution in the *bla*_TEM_ gene. All TEM-220 isolates were indistinguishable by NG-MAST.

**Conclusion:**

This is the first study which shows the prevalence of *bla*_TEM-220_ in *N. gonorrhoeae* isolates carrying Toronto/Rio plasmid in Argentina. Although the *bla*_TEM-220_ allele does not appear to be associated with an extended spectrum beta-lactamase (ESBL) phenotype of resistance, a single nucleotide polymorphism added to the *bla*_TEM-220_ or *bla*_TEM_ containing the T539C substitution could lead to the emergence of ESBL. Thus, it is imperative to investigate in surveillance programs, not only the plasmid type in PPNG isolates and the *bla*_TEM_ allele associated, but phenotypical characteristics and geographical distribution of isolates.

## Background

*Neisseria gonorrhoeae* is the etiological agent of the sexually transmitted infection gonorrhea, which remains a major public health issue. It represents 88 million of the estimated 448 million new cases of curable sexually transmitted infections that occur globally every year [1]. Over time, *N. gonorrhoeae* has progressively developed resistance to a wide range of antibiotics, including penicillin, ciprofloxacin, tetracyclines, macrolides, and more recently to third-generation cephalosporins [2, 3]. Antimicrobial resistance in *N. gonorrhoeae* became apparent shortly after the introduction of antibiotics into clinical practice. Moreover, its ability to acquire and/or maintain antibiotic resistance genes has become a considerable problem and an obstacle to successful therapeutic treatment [4, 5]. In 1943, penicillin was introduced as the first-line treatment of gonorrhea, but it was abandoned due to the emergence of chromosomally mediated penicillin resistance and PPNG isolates [6, 7]. PPNG strains were reported for the first time in 1976 in the United Kingdom and the United States [8, 9]. These strains produce a TEM type β-lactamase, which is carried on several related plasmids. To date, eight plasmid types have been described and named according to their epidemiological origin. Strains harbouring Asian, African and Toronto/Rio plasmids have been reported throughout the world [10, 11]. Other plasmid variants have been described, including Nimes, New Zealand, Johannesburg, and Australian plasmids [12–15]. The PPNG isolate in Argentina was reported in the 1980’s and has spread since then [16]. PPNG isolates are highly prevalent in our country and three plasmids types (Asian, African and Toronto/Rio plasmids) have been detected, resulting African and Toronto/Rio the most prevalent.

The first PPNG strains produced a TEM-1 β-lactamase, a class A enzyme encoded by *bla*_TEM-1_ allele [17]. TEM-1 β-lactamase efficiently hydrolyzes penicillins and many cephalosporins, but it is not an effective catalyst for extended spectrum cephalosporin turnover. For around thirty years, other β-lactamases were not described in gonococci. However, a PPNG isolate carrying a *bla*_TEM-135_ gene was reported from Thailand in the year 2009 [18]. TEM-135 was first described in 2005 in a *Salmonella enterica* subsp. *enterica* serovar Typhimurium isolate and differs from TEM-1 by one single nucleotide substitution at position 539 (T → C), resulting in a single amino acid substitution, M182T [19]. Prevalence studies from different countries revealed that PPNG isolates possessing Asian, Toronto/Rio, African and Australian plasmids carried *bla*_TEM-135_ gene [20–23]. Recently PPNG isolates carrying Toronto/Rio plasmid from Argentina that possessed the novel *bla*_TEM-220_ allele were identified [24]. TEM-220 differs from TEM-135 by one nucleotide substitution at position 547 (G → A), resulting in the amino acid substitution A185T. The prevalence and characteristics of TEM-220 strains worldwide are unknown and it seems imperative to study them.

Prevalence of PPNG isolates in Argentina has been reported previously, but national data of PPNG isolates possessing *bla*_TEM-220_ is lacking. The objective of our study was to detect *bla*_TEM-220_ gene in PPNG isolates possessing Toronto/Rio plasmid recovered between 2002 and 2011 in Argentina, and to evaluate the proportion of isolates producing non-TEM-220 containing the T539C substitution in the *bla*_TEM_ allele.

## Methods

### Collection of isolates

The *N. gonorrhoeae* clinical isolates investigated in this study were collected as part of Gonococcal Antimicrobial Susceptibility Surveillance Programme-Argentina (GASSP-AR). The isolates were obtained from 71 medical centers in 24 of the 24 Argentinian provinces from 2002 to 2011. We studied 151 PPNG isolates carrying Toronto/Rio plasmid previously identified as *N. gonorrhoeae* based on the characteristic colony morphology, Gram staining, oxidase test, superoxol test (30 % hydrogen peroxide), carbohydrate utilization test, and the Phadebact GC Monoclonal Test (MKL Diagnostic AB, Sollentuna, Sweden) [25]. All isolates were assigned a code number, preserved in tryptic soy broth (TSB) + 20 % glycerol at – 70 °C and added to the *N. gonorrhoeae* isolate collection of the reference laboratory. In this study only bacterial strains from clinical specimens collected from GASSP-AR were used and no patient information was accessed. Consequently, ethical approval was not required for this study.

Each frozen culture suspension was freshly subcultured on Difco GC Medium Base agar (BD, Franklin Lakes, NJ, USA) supplemented with 1 % Britalex enrichment supplement (Britania Lab., Argentina) and incubated at 35 °C in a humidified environment and enriched with 5 % CO_2_ during 24 to 48 hours. Cultures were examined once a day and colonies were subcultured using the same medium and incubation parameters as above. The identity of resurrected isolates was confirmed by Gram staining and the production of beta-lactamase by chromogenic Nitrocefin disc method (BD, Franklin Lakes, NJ, USA). Plasmid profile was studied using a boiling plasmid extraction method followed by agarose gel electrophoresis detection [26, 27].

### Antimicrobial susceptibility testing

The minimal inhibitory concentration (MIC) values of penicillin, ampicillin, cefuroxime, tetracycline, ciprofloxacin, ceftriaxone, cefixime and azithromycin were determined using the reference agar dilution method (CLSI) [28]. The *N. gonorrhoeae* ATCC 49226 and WHO reference strains were used as quality control for the MIC determinations [29]. The results were interpreted in accordance with CLSI breakpoints, except for azithromycin, for which the European Committee on Antimicrobial Susceptibility Testing (EUCAST) breakpoints were applied [30]. For ampicillin, as CLSI and EUCAST do not describe any breakpoints, the susceptibility categories were inferred from the penicillin breakpoints.

### DNA isolation

DNA was extracted from a fresh subculture by the boiling method. Colonies were suspended in 3 ml of ultrapure water and adjusted to a turbidity equivalent to a 1 McFarland standard using a calibrated turbidimeter. An aliquot of 500 μl of this suspension was transferred to a microcentrifuge tube with a capacity of 1.5 ml, then heated for 20 min at 98 °C, and cooled to 4 °C for 3 min. The mixture was centrifuged for 5 min at 10000 rpm and the supernatant was transferred to a new tube. The extracted DNA was used directly as template in the PCR and then stored at −20 °C.

### Mismatch amplification mutation assay - PCR

**Fig. 1:**
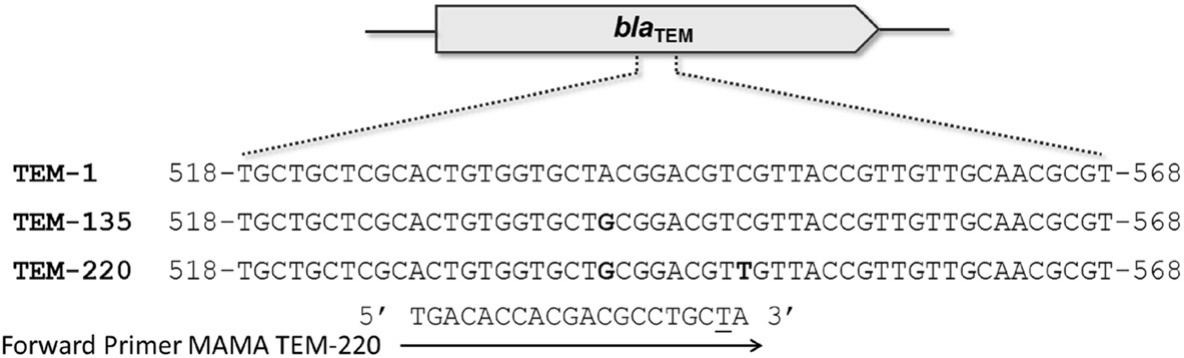
Alignment and difference of DNA sequence between the *bla*_TEM-1_, *bla*_TEM-135_, and *bla*_TEM-220_ gene. The nucleotide substitutions found in the different types of *bla*_TEM_ alleles are in bold type. The forward primer MAMA TEM-220 used in this study is shown schematically with arrows. Underlined letter indicates the nucleotide alteration near the 3′ end of the primer to enhance the 3′ mismatch effect

In order to detect *N. gonorrhoeae* strains possessing *bla*_TEM-220_ allele, a MAMA-PCR protocol was developed to identify a single nucleotide substitution at position 547 (G → A) in the *bla*_TEM_ gene. The MAMA TEM-220 primers used in this study were designed using free online Primer-BLAST (http://www.ncbi.nlm.nih.gov/tools/primer-blast/). Assay design consisted of a forward primer (T220-F, 5′-TGACACCACGACGCCTGCTA-3′), which annealed a *bla*_TEM-220_ allele-specific polymorphism, and a conserved reverse primer (T220-R, 5′-ATGATACCGCGAGACCCACG-3′). A ‘destabilizing’ mismatch at the penultimate base position from the 3′end of the forward primer was introduced to enhance the 3′ mismatch effect (Fig. 1) [31, 32]. Amplification was carried out in a 25 μl reaction mixture containing 1 μl DNA template, 1X reaction buffer, 1.0 mM MgCl_2_, 0.2 mM (each) deoxynucleoside triphosphate, 0.5 μM of each primer, and 0.75 unit of *Taq* polymerase (Invitrogen/Life Technologies, California, USA). The parameters of the amplification were as follows: initial denaturation at 96 °C for 2 min, followed by 30 cycles of 96 °C 10 s, 62 °C for 15 s, and 72 °C for 30 s, with final extension at 72 °C for 2 min in a MyCycler thermal cycler (Bio-Rad, California, USA). The amplification products were analyzed by means of electrophoresis on a 2 % (w/v) agarose gel. The specificity of the primer was evaluated by testing the previously described *N. gonorrhoeae* strains: IM4519 and IM4520 containing *bla*_TEM-220_, IM5923 and IM4540 containing *bla*_TEM-135_, and IM4629 and IM5257 containing *bla*_TEM-1_. These strains were used as a control in all PCRs.

### Detection of the T539C substitution in the *bla*_TEM_ gene

An allele-specific PCR method previously described was used to investigate *N. gonorrhoeae* isolates containing *bla*_TEM_ gene with the substitution at position 539 (T → C) [33]. *N. gonorrhoeae* strains IM4540 and IM5923 containing *bla*_TEM-135_ and IM4629 and IM5257 containing *bla*_TEM-1_ were used as controls in the PCR.

### Sequencing of the *bla*_TEM-220_ isolates

In all isolates that were positive to MAMA TEM-220 PCR, the whole *bla*_TEM_ was amplified and sequenced to confirm the *bla*_TEM-220_ allele. The *bla*_TEM_ allele was amplified as previously described [34]. The PCR products were purified using AccuPrep® PCR Purification Kit (Bioneer, Daejeon, Republic of Korea). DNA sequencing was performed using the BigDye® terminator v3.1 cycle sequencing kits (Applied Biosystems, Foster City, CA, USA) on an ABI 3500 Genetic Analyzer (Applied Biosystems, Foster City, CA, USA). The sequences were compared and aligned to previously described *bla*_TEM-220_ allele [GenBank: accession number KM998962] using the BioEdit (version 7.2.5) software.

### Genotyping

The NG-MAST analysis was performed exclusively on TEM-220-producing isolates. The *porB* and *tbpB* alleles were sequenced, and sequence types (STs) were assigned at the NG-MAST website (http://www.ng-mast.net) following the interpretative procedures previously described [35].

## Results

Over the period from 2002 to 2011, 3895 *N. gonorrhoeae* isolates were submitted from GASSP-AR for antimicrobial susceptibility testing. Overall, 476 (12.2 %) isolates were β-lactamase positive. The proportion of PPNG isolates was over 5 % in all years and ranged between 8.9 and 17.9 %. During the period of study two plasmid types were found. The African plasmid type was the most common in all years, comprising 325/476 isolates (68.3 %), followed by Toronto/Rio plasmid 151/476 (31.7 %). The serotyping of *N. gonorrhoeae* strains carrying Toronto/Rio plasmid revealed that the serogroup PorB1b (WII/III) predominated among these strains 134/151 (88.7 %), followed by serogroup PorB1a (WI) 17/151 (11.3 %).

### Use of the MAMA-PCR protocol for detection of the G547A substitution

**Fig. 2:**
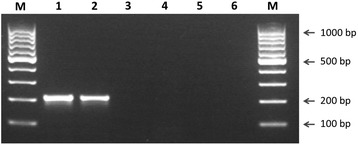
Discrimination of PPNG isolates with the G547A substitution in *bla*_TEM_ allele by using T220-f/T220-r primers. Lanes 1 and 2, indicate the 204 bp PCR products from strains IM4519 and IM4520 with the G547A mutation in *bla*_TEM_ allele; lanes 3, 4, 5 and 6 indicate PPNG control strains containing *bla*_TEM-1_ and *bla*_TEM-135_, respectively, without amplification; lane M, molecular size marker

A MAMA-PCR protocol was successfully designed for the identification of the substitution at position 547 (G → A) in the *bla*_TEM-220_ gene. Implementation of the corresponding PCR protocol showed that the primers were able to differentiate between PPNG strains with the G547A mutation in the *bla*_TEM_ allele and those without it (Fig. 2).

Four of the 151 PPNG isolates (2.6 %) showed a PCR product of the expected size (204 bp), suggesting that these isolates carried the *bla*_TEM-220_ gene. PCR amplification and sequencing analysis of the whole *bla*_TEM_ gene revealed a substitution at position 539 T → C and 547 G → A, confirming that these four isolates possessed the *bla*_TEM-220_ allele.

### Identification of the T539C substitution in the *bla*_TEM_ gene

The 147/151 MAMA TEM-220 PCR-negative isolates were investigated for the T539C substitution in the *bla*_TEM_ gene. From those, 137/147 (93.2 %) isolates showed a MAMA TEM-135 PCR product of the expected size (231 bp), which indicate the presence of the T539C substitution in the *bla*_TEM_ gene. The 10/147 (6.8 %) MAMA-TEM135 PCR-negative isolates, were TEM-1 PCR positive, suggesting the presence of *bla*_TEM-1_ allele in these isolates.

### MICs of Isolates

The MICs of 137 PPNG isolates carrying the T539C substitution in the *bla*_TEM_ gene and all (*n =* 4) PPNG isolates carrying *bla*_TEM-220_ allele are summarized in Table 1. All isolates were resistant to penicillin and ampicillin and susceptible to cefuroxime, cefixime and ceftriaxone. No differences in MICs of five beta-lactam antibiotics tested were observed in PPNG isolates producing TEM-220 and PPNG isolates producing non-TEM-220 carrying the T539C substitution in the *bla*_TEM_ gene. The 97.1 % of PPNG isolates containing the T539C substitution in the *bla*_TEM_ gene were susceptible to ciprofloxacin (MIC: ≤0.016 μg/ml), whereas 100 % of PPNG strains producing TEM-220 were susceptible. All isolates were susceptible to azithromycin (MIC: ≤ 0.25 μg/ml).

**Table 1:** Susceptibility of four PPNG isolates producing TEM-220 β-lactamase and PPNG strains containing the T539C substitution in the *bla*_TEM_ allele

			MIC (μg/ml)			
Antimicrobial agent	^a^T539C		TEM-220			
	^b^MIC_50_	^c^MIC_90_	Isolate 1	Isolate 2	Isolate 3	Isolate 4
Penicillin	64	128	32	64	32	64
Ampicillin	128	256	64	128	128	128
Cefuroxime	0,25	0,5	0,125	0,125	0,125	0,125
Cefixime	0,016	0,016	0,008	0,008	0,008	0,008
Ceftriaxone	0,004	0,008	0,008	0,004	0,008	0,004
Tetracycline	1	4	0,5	1	1	1
Ciprofloxacin	0,004	0,008	0,002	0,004	0,004	0,004
Azithromycin	0,25	0,25	0,25	0,25	0,25	0,25

^a^Number of PPNG isolates containing the T539C substitution (*n =* 137)

^b^MIC_50_ : MIC of inhibition of 50 % of strains

^c^MIC_90_: MIC of inhibition of 90 % of strains

### Genotyping

The four PPNG isolates possessing *bla*_TEM-220_ were isolated in 3 of the 24 provinces of Argentina. The geographic distribution of these isolates was in the north and southern part of the country. All of this isolates were indistinguishable by NG-MAST and shared alleles *por* 6406 and *tbpB* 21, which make up NG-MAST type 10972.

## Discussion

This study was conducted to analyze the prevalence of *bla*_TEM-220_ and to detect the T539C substitution in the *bla*_TEM_ allele in PPNG isolates carrying Toronto/Rio plasmid. Prevalence data on plasmid profile and TEM-type beta-lactamase of PPNG isolates from South America is limited [36]. In Argentina, penicillin is not recommended as first-line therapy for the treatment of gonorrhea since the early 1990s [37]. However, a high percentage of isolates are beta-lactamase positive. In this study, the prevalence of PPNG strains was 12.2 % between 2002 and 2011 and the plasmid profile revealed two types of circulating plasmids, resulting the African-type plasmid the most frequent in all years.

PPNG isolates possessing *bla*_TEM-220_ have recently been described [24]. Here, we describe a MAMA PCR method for the specific detection of the G547A substitution in the *bla*_TEM-220_ allele. The results showed that the MAMA PCR successfully detected four PPNG isolates carrying *bla*_TEM-220_, which were confirmed by sequencing the whole *bla*_TEM_ gene. The *bla*_TEM-220_ allele was found in 2.6 % of all isolates. These strains were resistant to penicillin and ampicillin, but susceptible to cefuroxime, cefixime and ceftriaxone. According to this, it is believed that these substitutions have no effect on the substrate spectra of the enzyme. Moreover, the β-lactamase phenotype of *bla*_TEM-220_ gene does not correspond to an ESBL phenotype. All isolates were assigned to serogroup PorB1b (WII/III) and NG-MAST was used to investigate the diversity and relatedness of the four TEM-220-producing isolates. All TEM-220 isolates were assigned the same ST 10972, indicating that these strains had originated from a common ancestor. In a previous study of *bla*_TEM-135_ possessing isolates in Argentina, ST 10972 was found to be prevalent in 19.0 % of the TEM-135 isolates carrying Toronto/Rio plasmid [24]. We did not observe different patterns of resistance when comparing susceptibility or resistance to penicillin, cefixime, ceftriaxone, ciprofloxacin, tetracycline and azithromycin between TEM-220 isolates and previously described TEM-135 isolates with ST 10972 (data not shown). Based on these observations, other factor(s) than antimicrobial selective pressure may have been the selective force that drove the emergence of TEM-220 beta-lactamase. Although *bla*_TEM-220_ allele might have been acquired through horizontal gene transfer from an unknown bacterial source, the highly similar contexts between TEM-135 and TEM-220 isolates suggest that *bla*_TEM-220_ allele could have evolved from *bla*_TEM-135_ gene by a single nucleotide polymorphism (SNP).

The study of the substitution at position 539 (T → C) in MAMA TEM-220 PCR-negative isolates revealed that 93.2 % of the isolates contained this mutation in the *bla*_TEM_ gene. The T539C mutation results in a single amino acid substitution (M182T), which is believed to restore the stability of the enzyme affected by substitutions near the active site [38, 39]. M182T accompanies additional mutations that may extend the substrate specificity of the enzyme to ESBL such as TEM-20 and TEM-52 [40, 41]. Nowadays, extended spectrum cephalosporins are used as first line therapy in our country, and resistance has not been detected. However, an additional SNP added to the *bla*_TEM-220_ or *bla*_TEM_ containing M182T substitution could lead to the emergence of ESBL as a response to the selective pressure induced by extended spectrum cephalosporin. This leads to the imperative need for monitoring for the possible emergence and spread of ESBL-producing *N. gonorrhoeae*.

A limitation of the current study was the lack of demographic and epidemiological information which limited the comparison between PPNG isolates and patient characteristics.

## Conclusions

In summary, this study shows the prevalence of TEM-220 in *N. gonorrhoeae* isolates carrying Toronto/Rio plasmid in Argentina. The *bla*_TEM-220_ allele does not appear to be associated with an ESBL phenotype, and high percentage of PPNG isolates carrying Toronto/Rio plasmid contained the T539C substitution in the *bla*_TEM_ gene. Knowledge about the diversity and prevalence of β-lactamases in PPNG strains is crucial in order to detect the possible emergence of resistant variants to extended-spectrum cephalosporin.
